# Hemodynamic Factors Driving Peripheral Chemoreceptor Hypersensitivity: Is Severe Aortic Stenosis Treated with Transcatheter Aortic Valve Implantation a Valuable Human Model?

**DOI:** 10.3390/biomedicines13030611

**Published:** 2025-03-03

**Authors:** Maksym Jura, Stanisław Tubek, Jędrzej Reczuch, Rafał Seredyński, Piotr Niewiński, Marcin Protasiewicz, Beata Ponikowska, Bartłomiej Paleczny

**Affiliations:** 1 Department of Physiology and Pathophysiology, Wroclaw Medical University, Chałubińskiego 10, 50-368 Wroclaw, Poland; maksym.jura@umw.edu.pl (M.J.); rafal.seredynski@umw.edu.pl (R.S.); beata.ponikowska@umw.edu.pl (B.P.); 2 Institute of Heart Diseases, Wroclaw Medical University, Borowska 213, 50-556 Wroclaw, Poland; s.tubek@wp.eu (S.T.); jedrzej.reczuch@gmail.com (J.R.); piotr.niewinski@umw.edu.pl (P.N.); marcin.protasiewicz@umw.edu.pl (M.P.)

**Keywords:** peripheral chemoreceptors, peripheral chemoreflex, carotid body, aortic body, aortic stenosis, transcatheter aortic valve implantation

## Abstract

**Background:** A reduction in carotid artery blood flow (CABF) and ultimately in wall shear stress (WSS) is a major driver of heightened peripheral chemoreceptor (PCh) activity in animal models of heart failure. However, it is yet to be translated to humans. To provide more insight into this matter, we considered severe aortic stenosis (AS) before and after transcatheter aortic valve implantation (TAVI) as a human model of carotid and aortic body function under dramatically different hemodynamic conditions. **Materials and Methods:** A total of 26 severe AS patients (aged 77 ± 6 y, body mass index: 29.1 ± 5.1 kg/m^2^, left ventricular ejection fraction (LVEF): 50 ± 15%) were subjected to a transient hypoxia test twice: immediately before vs. 1–4 months after TAVI (median follow-up: 95 days). PCh function was analyzed in terms of ventilatory (HVR, L/min/SpO_2_%) and heart rate responses to hypoxia (HR slope, bpm/SpO_2_%). Standard ultrasound (inc. aortic valve area [AVA], mean aortic valve gradient, peak aortic jet velocity, LVEF, and CABF), respiratory, hemodynamic, and blood parameters were collected at both visits. Pre- vs. post-TAVI data regarding HVR and HR slopes were available for N = 26 and N = 10 patients, respectively. **Results:** HVR did not change following TAVI (pre- vs. post-TAVI: 0.42 ± 0.29 vs. 0.39 ± 0.33 L/min/SpO_2_%, *p* = 0.523). The HR slope increased after TAVI (pre- vs. post-TAVI: 0.26 ± 0.23 vs. 0.37 ± 0.30 bpm/SpO_2_%, *p* = 0.019), and the magnitude of the increase was strongly associated with an increase in AVA (Spearman’s R = 0.80, *p* = 0.006). No other significant relations between pre- vs. post-TAVI changes in PCh activity measures vs. hemodynamic parameters were found (all *p* > 0.12). **Conclusions:** The ventilatory component of the PCh reflex (defined as HVR) in severe AS patients is not affected by TAVI, and pre-TAVI values in this group are fairly comparable to those reported previously for healthy subjects. On the contrary, HR responses to hypoxia are increased after TAVI, and pre-TAVI values appear to be lower compared to the healthy population. An extraordinarily strong correlation between post-TAVI increases in HR slope and AVA may suggest that hemodynamic repercussions of the surgery in the aortic body area (most likely reduced WSS) play a critical role in determining aortic body function with a negligible effect on the carotid bodies. However, caution is needed when interpreting the results of the HR response to hypoxia in our study due to the small sample size (N = 10).

## 1. Introduction

Two populations of chemosensitive units situated in the carotid bifurcation (carotid bodies) and on the aortic arch (aortic bodies) are the main peripheral chemoreceptors (PCh) in mammals. The carotid and aortic bodies are critically involved in orchestrating the cardio-respiratory response to hypoxia and hypercapnia under acute and chronic scenarios [1,2,3,4]. Functional distinctions between the carotid and aortic bodies appear to exist, with the former underlying ventilatory responses and the latter underlying tachycardic responses [5,6,7,8]. While traditionally associated with O_2_ and CO_2_ homeostasis, PCh sense numerous physicochemical stimuli [9], with decreased perfusion being one of them [10,11].

PCh hypersensitivity was found to accompany certain sympathetically mediated disorders, particularly heart failure (HF) [12,13] and essential hypertension [14,15]. Worse clinical outcomes in HF patients displaying PCh hypersensitivity were reported more than two decades ago by Ponikowski et al. [12] and have persisted over time, as demonstrated recently in a large cohort of 369 patients receiving optimal guideline-indicated therapy [16]. In addition, animal studies provide compelling evidence for the causal nature of this relationship [17,18], which is most likely driven by PCh contribution to sympathetic overactivation [19]. Surgical and pharmacological approaches to peripheral chemoreflex deactivation for the treatment of HF and resistant hypertension have been proposed recently [8,15,20,21], thereby emphasizing the clinical potential and hopes associated with modulation of the peripheral chemoreflex.

The substantial interest in PCh deactivation is in stark contrast to the scarcity of human studies on the mechanisms underpinning PCh hypersensitivity. However, results from animal models provide valuable insight into this matter [22,23,24,25,26], showing that (1) replicating the trajectory of HF-associated carotid flow reduction in healthy animals results in peripheral chemoreflex augmentation similar to that found in the HF group [23,24]; (2) PCh hypersensitivity is present in low-output HF models but not in high-output HF models [25]; (3) carotid flow reduction dampens the expression of the shear-stress-sensitive transcription factor Krüppel-like factor 2 (KLF2) [26]; and (4) selective restoration of KLF2 expression in the carotid bodies of HF animals (via viral transfection) normalizes PCh function [26]. In light of this, limited blood flow in the vicinity of carotid/aortic bodies resulting in wall shear stress (WSS) reduction emerges clearly as the main factor driving PCh hypersensitivity in HF, although this has yet to be verified in human studies.

In pursuing a clinical model of acutely reversible low vs. high flow and/or low vs. high WSS in the aortic and carotid regions, we have focused our attention on patients with severe aortic stenosis (AS) referred for transcutaneous aortic valve implantation (TAVI). AS stems from the progressive thickening, fibrosis, and calcification of the leaflets, which reduces the valve opening and obstructs left ventricular outflow during ventricular systole. As a result, blood flow from the heart through the stenotic valve into the aorta (and possibly large arteries downstream, including the carotid arteries) is markedly altered. Unlike HF, the core abnormality underlying depressed cardiac function in severe AS is readily corrigible through surgical aortic valve replacement (SAVR) or transcatheter aortic valve implantation (TAVI) [27,28,29,30]. Both methods have been demonstrated to increase left ventricular ejection fraction (LVEF) or stroke volume [31,32,33,34], whereas the results regarding a possible increase in carotid artery blood flow (CABF) are variable [33,35,36].

**Figure 1:**
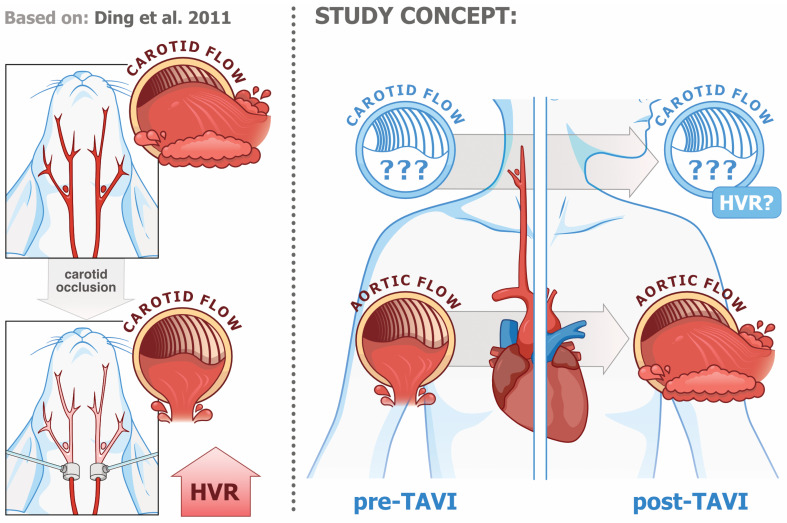
Visualization of the study concept [24].

It appears justified to assume that both severe AS and TAVI affect the function of the carotid and aortic bodies by changing the hemodynamic environment of these structures. Given that the anatomical distance to the aortic valve is greater for carotid bodies and lower for aortic bodies, the reflex responses from the latter should be affected to a greater extent. Figure 1 illustrates the concept of this study.

In this study, PCh function in severe AS patients has been assessed twice: before and 1–4 months after the TAVI surgery. Ultimately, this study was expected to assess the feasibility and rationale for using severe AS treated with TAVI as a human model of PCh function under low vs. high blood flow and/or WSS conditions.

## 2. Methods

### 2.1. Study Population and Ethical Approval

Between November 2021 and June 2022, all patients with severe symptomatic AS who were evaluated at the Institute of Heart Diseases, University Hospital in Wroclaw (Poland) by a Heart Team for their eligibility for TAVI and fulfilled the inclusion criteria of this study were asked to participate. The exclusion criteria were inability to comply with the protocol and HF with a reduced ejection fraction if an improvement in LVEF is not likely (i.e., due to a significant scar burden).

The study protocol was approved by the local Ethics Committee (the Bioethics Committee of Wroclaw Medical University, permission no. KB-827/2021). All participants provided written informed consent. This study conformed to the standards set by the Declaration of Helsinki, except for registration in a database.

### 2.2. Study Protocol

The protocol of this study included: (i) an assessment of PCh function with the transient hypoxia test, (ii) the measurement of CABF with ultrasonography, and (iii) a standard echocardiographic examination and routine laboratory tests. The assessments were performed within 6 weeks prior to the surgery and repeated within 9 months after TAVI. The baseline period preceding the transient hypoxia test was used to calculate resting values of respiratory and haemodynamic variables, as the average of 10 min of the recording.

All experiments were conducted at the Institute of Heart Diseases, University Hospital in Wroclaw. In particular, all experiments including transient hypoxia tests were conducted by adequately trained operators under the continuous supervision of a cardiologist, with continuous and real-time monitoring of cardiovascular and respiratory parameters.

### 2.3. Transcatheter Aortic Valve Implantation

The retrograde transfemoral approach was used as the standard access site for TAVI, and the alternative approaches (transcarotid, aortic, and subclavian) were considered in patients with unsuitable femoral access. The procedures were conducted in a catheterization laboratory, with patients under general anesthesia employing fluoroscopy and transesophageal echocardiography guidance.

### 2.4. Assessment of Peripheral Chemoreflex Function with the Transient Hypoxia Test

For the test procedure, an examination was performed with the patient lying supine in a quiet, light-attenuated room, preferably in the morning (between 07:00 and 12:00). The transient hypoxia test was employed for the assessment of PCh function, as described elsewhere [13,37,38]. In brief, the test relies on repeated bouts of hypoxia produced by the administration of pure nitrogen into the breathing circuit. The test comprises 4–12 nitrogen administrations lasting 5–40 s each and separated with ≥3 min periods of breathing with room air. The first administration lasted 5 s and the length of subsequent administrations was increased gradually until a nadir SpO_2_ of ~70% was achieved or until a clear-cut and reproducible increase in instantaneous minute ventilation was achieved. Then, the length of the following administrations was randomly modified within the previously identified range in order to obtain a wide range of SpO_2_ nadirs, but without exposing the patient to excessive, difficult-to-tolerate discomfort [39].

During the test, the patient breathed through an oronasal mask (7450 series V2 mask, Hans Rudolph, Inc., Shawnee, KS, USA) connected to a T-shaped two-way non-rebreathing valve (Hans Rudolph, Inc.). A one-meter-long breathing tube was mounted on the inspiratory side of the valve and opened to room air. Nitrogen from the tank was administrated directly into the breathing tube at the inspiratory side of the breathing circuit via a small-diameter flexible pipe. The expiratory arm of the valve was connected, with a breathing tube, to a 1000 L/min flow head of a differential pressure transducer (FE141 Spirometer, ADInstruments, Dunedin, New Zealand) enabling the continuous measurement of expiratory airflow. Tidal volume (VT, L) and breathing rate (BR, breaths/min) were calculated from the expiratory flow for each breath and used to estimate instantaneous minute ventilation ($\dot{V}$, L/min). Blood oxygen saturation (SpO_2_, %) was measured with a pulse oximeter (Radical-7, Masimo Corporation, Irvine, CA, USA) and a probe placed on the patient’s earlobe. End-tidal CO_2_ (etCO_2_, mmHg) was recorded with a capnograph (Capstar 100, CWE, Ardmore, PL, USA).

Hemodynamic data (systolic blood pressure, SBP, mmHg; diastolic blood pressure, DBP, mmHg; cardiac output, CO, L/min; and systemic vascular resistance, SVR, dyn × s/cm^5^) were collected in a continuous and non-invasive manner using the Nexfin monitor (BMEYE, Amsterdam, The Netherlands). Heart rate (HR, bpm) was calculated based on the ECG signal (BioAmp, ADInstruments, Dunedin, New Zealand).

All data were acquired at a sampling frequency of 1 kHz with the use of a data acquisition system (PowerLab 16/30, ADInstruments, Dunedin, New Zealand) and stored on a laptop computer.

### 2.5. Quantification of Peripheral Chemoreflex Sensitivity

Ventilatory response: A detailed description of the computational procedure has been published elsewhere [13,39]. In brief, hypoxic ventilatory response (HVR, L/min/SpO_2_%) was defined as a slope of the regression line fitting the points obtained by plotting pre- and post-hypoxic SpO_2_ against corresponding $\dot{V}$ values. Specifically, for each N2-induced hypoxic episode, two points were obtained: (i) the pre-hypoxic point, indicated by plotting the average SpO_2_ from a 60 s beat-to-beat SpO_2_ signal preceding the hypoxic episode against the average $\dot{V}$ from the corresponding time window, and (ii) the post-hypoxic point, calculated by plotting the SpO_2_ nadir following N2 administration (within one minute after the end of N2 administration) against the average of the three largest consecutive $\dot{V}$ values (referring to three consecutive breaths) within a time window starting 5 heartbeats before the SpO_2_ nadir and ending 20 heartbeats after the SpO_2_ nadir.

Heart rate response: Ectopic beats in the ECG signal localized within the analysis window have been removed using the Beat Classifier View module of LabChart (ADInstruments, Dunedin, New Zealand). Subsequently, the HR signal was averaged over three heartbeats and converted to beat-to-beat data before analysis.

The procedure analogous to that described above for HVR was employed to quantify the HR response to transient hypoxia (HR slope, bpm/SpO_2_%) with beat-to-beat HR data used in place of $\dot{V}$ data. In contrast to the HVR calculation, a single highest HR value was used to calculate the post-hypoxic point for each hypoxic episode.

### 2.6. Ultrasound Measurement of Carotid Blood Flow

Test procedure: A method similar to that described by Sato et al. (2011) [40] was employed for CABF estimation. The brightness mode was used to obtain short video recordings of the carotid common artery in a longitudinal section, about 2 cm distal to the bulb. Several videos (2–4, typically) were collected for each carotid artery of the same patient. Once the B-mode videos of a given artery had been obtained, the pulse wave mode was used to measure blood flow velocity with the Doppler velocity spectrum. The blood velocity measurement was based on a 4 s time window.

When performing blood flow velocity measurements, care was taken to maintain an insonation angle of 60° or less. All the carotid ultrasonography measurements were conducted by the same sonographer with extensive clinical experience (S.T.).

Quantification of CABF: Systolic and diastolic diameters of the carotid artery were measured from B-mode videos using the Tracker software (v. 6.1.1, https://opensourcephysics.github.io/tracker-website/, accessed on 21 February 2025, Aptos, CA, USA) and following the methodology outlined by Wikstrand (2007) [41], and calculated in relation to the blood pressure curve according to the following formula: mean diameter = (systolic diameter × 1/3) + (diastolic diameter × 2/3). Values of mean diameter calculated from multiple B-mode videos obtained from the same carotid artery were averaged. The time-averaged mean flow velocity (TAMEAN, m/s) retrieved from the Doppler velocity spectrum was taken as a measure of mean blood flow velocity in the carotid artery.

Finally, CABF was calculated by multiplying the cross-sectional area of the carotid artery (π × (mean diameter/2)2) by the mean blood flow velocity in the carotid artery (TAMEAN), according to the following formula: CABF = (π × (mean diameter/2)2) × TAMEAN × 60 (ml/min).

### 2.7. Echocardiography and Laboratory Tests

Standard transthoracic echocardiography and assessment of AS severity was performed according to guideline recommendations [42,43,44]. The mean aortic valve gradient (mmHg) was calculated using the simplified Bernoulli equation, the aortic valve area (AVA, cm^2^) was estimated with the continuity equation, the aortic jet velocity (m/s) was measured using a continuous-wave Doppler ultrasound from the acoustic window that provided the highest velocity, and the left ventricular ejection fraction (LVEF, %) was determined using the biplane Simpson method. Routine laboratory tests were performed, including blood hemoglobin (g/dL), hematocrit (%), creatinine (mg/dL), sodium (mmol/L), potassium (mmol/L), and N-terminal pro-B-type natriuretic peptide (NT-proBNP, pg/mL).

### 2.8. Statistical Analysis

Normal Gaussian distributions of the continuous variables were assessed with the Kolmogorov–Smirnov test. Continuous data are presented as mean ± standard deviation (SD, for data with normal distribution) or median with interquartile range (IQR, for data with non-normal distribution). Categorical variables are presented as numbers and percentages. Student’s paired *t*-test or the Wilcoxon test was used for within-subject comparisons. Associations between variables were assessed using Spearman’s rank correlation coefficient.

In order to compare pre-TAVI values of the HVR and HR slopes with the literature data, a paired *t*-test was used for the studies reporting mean ± SD. Studies presenting data as median with quartiles were excluded from the analysis.

Statistica v.13.3 (StatSoft, Tulsa, OK, USA) and MATLAB R2021b (Mathworks, Natick, MA, USA) were used for statistical analysis. A *p*-value < 0.05 was considered statistically significant.

## 3. Results

### 3.1. Study Population

A total of 34 patients with severe AS were consecutively recruited for this study. Out of these, 26 (76%) were re-examined following TAVI, whereas the remaining refused to undergo a post-TAVI examination (N = 8). Thus, the final sample size for the present study was N = 26.

Furthermore, data from 16 patients have been excluded from the analysis of the HR response to hypoxia due to the presence of one or more of the following: (i) decreased beta blocker dosage before the post-TAVI assessment (N = 10), (ii) atrial fibrillation during the measurement (N = 5), or (iii) pacemaker implantation after the TAVI surgery (N = 3). Thus, the final sample for HR slope analysis was N = 10. CABF was collected in a subgroup of 18 patients.

Baseline characteristics for the whole group (N = 26) are presented in Table 1. The transfemoral access method was used for all but two patients, for whom aortic (N = 1) and transcarotid (N = 1) access was utilized. The time between the pre-TAVI assessment of PCh function using the transient hypoxia method and the surgery was 1 day in 21 (81%) patients and ranged between 4–36 days for the remaining patients. The post-TAVI assessment of PCh function was performed after a median of 95 days (IQR: 42–108 days). The pre-TAVI examination of CABF with Doppler ultrasonography was conducted on the day of TAVI surgery in 16 (89%) patients, and 2 and 7 days before TAVI in the two remaining subjects. Post-TAVI examination of CABF was conducted after a median of 93 days (IQR: 42–108 days).

### 3.2. The Effect of TAVI on Clinical Parameters

Changes in clinical parameters after the surgery followed a well-documented pattern [45]. Ultrasound examination revealed an increase in the aortic valve area and LVEF, and a decrease in the mean aortic valve gradient and peak aortic jet velocity (all *p* < 0.03, Table 2). CABF remained virtually unchanged following TAVI (*p* = 0.933, Table 2). Baseline respiratory and hemodynamic measures did not change after the surgery (all *p* > 0.10, Table 2). Blood hemoglobin and hematocrit increased, while creatinine and NT-proBNP levels decreased after TAVI (all *p* < 0.05, Table 2).

### 3.3. The Effect of TAVI on Peripheral Chemoreflex Function

**Figure 2:**
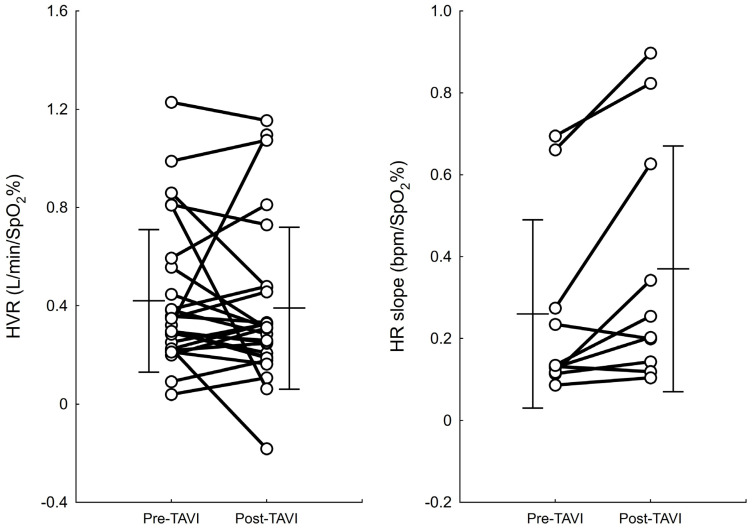
Individual data on the HVR and HR slope measured before vs. after TAVI. Bars and whiskers indicate mean ± standard deviation (SD).

**Figure 3:**
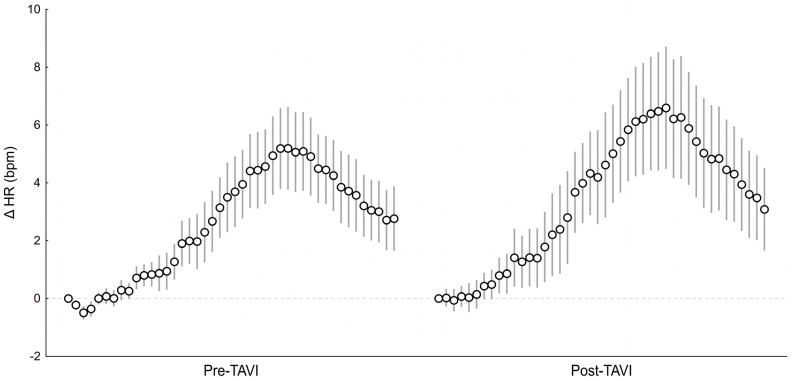
The averaged HR response to transient hypoxia before vs. after TAVI (beat-to-beat data). Bars and whiskers indicate the mean ± standard error of measurement (SEM).

The ventilatory component of the peripheral chemoreflex remained unaltered following the surgery (pre- vs. post-surgery HVR: 0.42 ± 0.29 vs. 0.39 ± 0.33 L/min/SpO_2_%, *p* = 0.523), whereas the HR response to hypoxia was increased after TAVI (pre- vs. post-surgery HR slope: 0.26 ± 0.23 vs. 0.37 ± 0.30 bpm/SpO_2_%, *p* = 0.019, Figure 2). Figure 3 shows the average beat-to-beat HR response to hypoxia before and after the surgery. For better readability, the standard error of measurement (SEM) was shown instead of SD.

### 3.4. Peripheral Chemoreflex Function Before TAVI as Compared with the Literature Data

**Figure 4:**
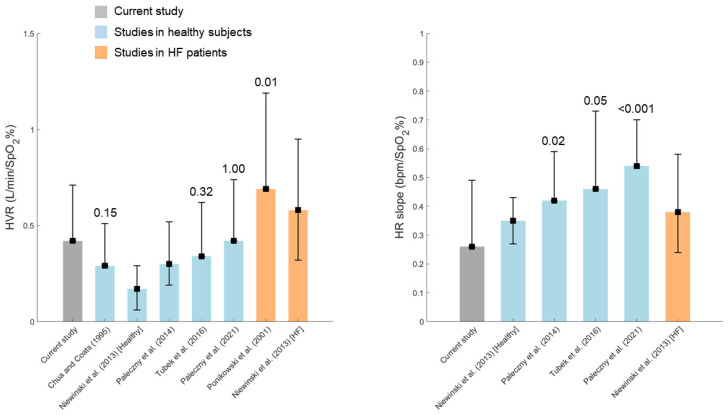
Pre-TAVI measures of HVR and HR slopes in the current study as compared with the literature data. Squares with upper whiskers only indicate the mean and SD, respectively. Squares with lower and upper whiskers indicate the median and lower and upper quartiles, respectively. *p*-values for comparisons with paired Student’s *t*-tests vs. the current study are shown above the whiskers. Statistical comparisons were conducted for studies reporting mean and SD [7,12,13,38,46,47].

Figure 4 shows the comparison of the pre-TAVI values of HVR and HR slopes obtained in the examined group and the values from previous studies employing the transient hypoxia method. In order to minimize the possible effect of age-related changes, only studies with healthy subjects/HF patients aged 50 years or older were included in the analysis. HVR values in the current study were comparable to those reported in the other studies for healthy subjects, and statistically lower than the values found by Ponikowski et al. (2001) [12] in HF patients. On the other hand, HR slope values obtained in the current study were lower than the values observed in a few studies in healthy subjects. Please note that differences between the current study and studies using medians with quartiles were not tested.

There are correlations between pre- vs. post-surgery changes in selected hemodynamic parameters and the change in peripheral chemoreflex function.

**Figure 5:**
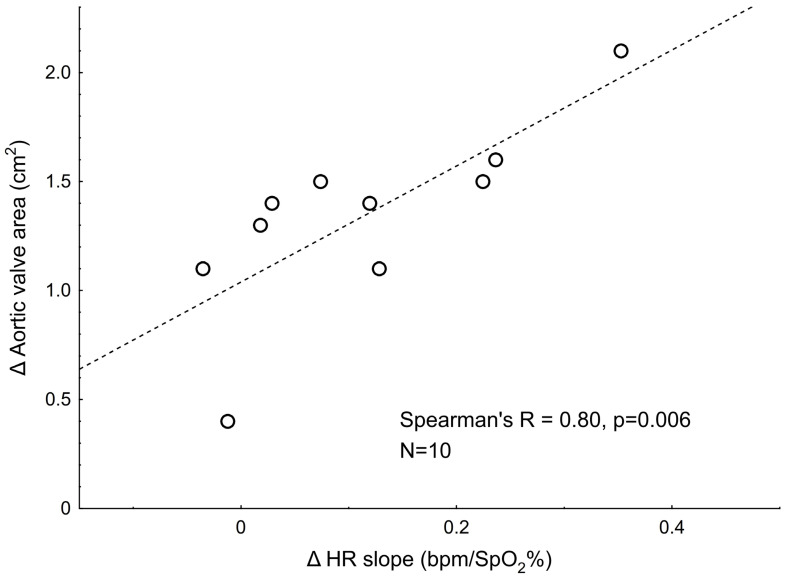
Scatter plot illustrating the relationship between pre- vs. post-surgery changes in HR slope and the change in aortic valve area.

The post-TAVI increase in the aortic valve area was correlated positively to changes in HR response to hypoxia (Table 3, Figure 5). No other statistically significant correlations have been found for the changes in HVR or HR slopes and the changes in analyzed clinical parameters (all *p* > 0.13, Table 3).

## 4. Discussion

Despite compelling evidence from animal studies indicating that peripheral chemoreceptor hyperactivity in HF is primarily driven by reduced blood flow through the carotid body [23,24,25,26], this issue has not been investigated in humans so far. However, instead of focusing on the HF population, we have attempted to examine PCh function in patients with severe AS before and after TAVI surgery. Severe AS is accompanied by deteriorated left ventricular outflow (similarly to HF) [27,28], but unlike in HF, the core abnormality in this disorder is readily corrigible by means of aortic valve replacement. Therefore, it may be considered a relatively “pure” and reversible model of reduced aortic and/or carotid body blood flow [32,48,49].

The key findings from this study are that (i) there was no change in HVR post-TAVI, (ii) an enhanced HR response to hypoxia was observed post-TAVI, and (iii) there is a robust positive correlation between the post-TAVI increase in the aortic valve area and HR response potentiation (Spearman’s R = 0.80). Given that HVR is most likely mediated via carotid bodies, whereas the cardiac response stems from the stimulation of aortic bodies, it is tempting to speculate that the anatomical distance to the aortic valve (substantially smaller for the aortic bodies as compared with the carotid bodies) underlies this discrepancy. The relation between post-TAVI changes in the aortic valve area and the HR response provides further support for this explanation.

### 4.1. TAVI Does Not Affect the Ventilatory Component of the Peripheral Chemoreflex

We found no difference between pre- vs. post-TAVI measurements of HVR in our population. Several aspects of these results merit comment. First, the PCh sensitivity and HVR have never been studied in men, and the rationale for expecting a heightened HVR in severe AS patients stems from animal studies demonstrating that the chronic reduction of carotid artery blood flow (CABF) results in the progressive sensitization of PCh [22,23,24]. However, the HVR in our population was virtually the same as that reported for healthy individuals in earlier studies from our center (i.e., median HVR: 0.30 (0.19–0.52) L/min/SpO_2_% in men aged 50 years or older in Paleczny et al. [38]) or in studies from other research groups [37,46,50,51], and markedly lower than the values found typically for HF patients [12,13].

Normal PCh function in severe AS appears surprising, given that the signs of autonomic derangement (sympathovagal imbalance with sympathetic predominance and vagal withdrawal) found typically in HF [16,52] have also been reported in severe AS patients, as evidenced by microneurography [53] and ECG-based measures such as heart rate turbulence [54,55,56], heart rate variability analysis [54,57,58], and deceleration capacity [55,56,59].

The absence of changes in hypoxic ventilatory response (mediated via carotid bodies) following TAVI, accompanied by an enhanced heart rate response to hypoxia (mediated most likely via aortic bodies), may suggest that hemodynamic repercussions of the valve replacement are strong enough to influence the chemosensitive areas situated in the immediate vicinity of the valve (namely aortic bodies), but not the more distant ones (carotid bodies). In line with this, carotid artery blood flow (CABF) did not change after the surgery in our population. Such results corroborate the most recent study by van Houte et al. [33], who reported no change in carotid common artery blood flow when re-examined 10 min after successful valve implantation in a group of 25 patients (mostly in NYHA class III [88%], with LVEF > 50% [80%]). However, this is in contrast to other reports. Cammalleri et al. [36] found a ~70% increase in the left internal carotid artery flow immediately after the completion of the procedure in a group of 62 patients (mostly in NYHA classes III/IV [87%], LVEF mean ± SD: 49 ± 11%), and the results were virtually the same between the subgroups of patients with LVEF ≤ 40 vs. >40%. A slight increase in CABF (~3%) was found by Kleczyński et al. [35]. The study enrolled 30 carefully selected patients with isolated AS, with preserved LVEF (>50%), without atrial fibrillation, and taking only β-blockers. Differences between the studies regarding the clinical status of the examined populations, along with certain methodological discrepancies (see discussion in van Houte et al. [33]), likely contribute to the conflicting results. The population examined in the current study is similar to that studied by Cammalleri et al. [36].

However, it must be underlined that, according to the current understanding, a reduction in WSS, and not the blood flow per se, potentiates chemoreceptor responsiveness. We did not measure WSS in our population. However, decreased WSS in the carotid common artery, accompanied by well-preserved CABF, has been reported in AS patients referred for aortic valve replacement. Of note, the surgery improved WSS substantially in that study, resulting in favorable remodeling of the carotid artery wall [49]. In brief, the lack of changes in CABF does not necessarily preclude the presence of hemodynamic factors capable of affecting peripheral chemoreceptor activity.

Lastly, the computational study by Ardakani et al. [60], utilizing a handful of ideal (hypothetical) as well as realistic (reconstructed from cadaveric data) geometries of the carotid bifurcation, has revealed that WSS is higher at the bifurcation (rostrally to the carotid bulb)—the most prevalent site of the carotid body [61]—as compared with the remaining area of the bulb. Therefore, one cannot preclude that certain anatomic peculiarities of the human carotid artery ensure the preservation of WSS in the carotid body area (and thereby, PCh function) within a wide range of CABF.

In summary, both normal PCh function before TAVI and the absence of changes in the ventilatory response to hypoxia after the surgery call into question the putative utility of severe AS treated with TAVI as a clinical model of peripheral chemoreceptor function under low- vs. high-blood-flow conditions.

### 4.2. Heart Rate Response to Hypoxia Is Suppressed in Severe Aortic Stenosis with Partial Restoration Post-TAVI

While the ventilatory component of the peripheral chemoreflex appears to be mediated via the carotid bodies, as evidenced by the attenuation of the response after bilateral carotid body removal in HF patients [5], the physiological background of the HR response remains vague [6,39,62,63,64]. Early studies on anesthetized and artificially ventilated animals have found that the isolated stimulation of carotid and aortic bodies results in bradycardia and tachycardia, respectively [63], and such opposite responses subsequently received further support from human studies [7].

Pre-TAVI measures of the heart rate response to hypoxia (HR slope: 0.26 ± 0.23 bpm/SpO_2_%) in the examined group were lower than the values reported for healthy subjects in previous studies from our research team (i.e., mean ± SD: 0.42 ± 0.17 bpm/SpO_2_% in ref. [38] for healthy men > 50 years of age; median and IQR: 0.35 (0.27–0.43) bpm/SpO_2_% in ref. [13] for healthy subjects aged 54 ± 6 years). Differences in the participants’ age between studies likely contributed to such results, as a gradual decline in the cardiac response to hypoxia with aging has been demonstrated previously [65].

However, the HR slope increased after TAVI, suggesting that certain AS-related factors must restrain the cardiac acceleratory potential before the surgery. Following the reasoning presented by Ding et al. [23,24] in the animal model of peripheral chemoreceptor sensitization generated by CABF restriction, we rather expected the HR hypoxic response to be heightened before TAVI (due to decreased blood flow through the aortic bodies, resulting in decreased WSS in this area) and reduced/normalized after TAVI. However, importantly, Tan et al. [66] have shown recently, using a combination of magnetic resonance imaging and computational flow simulations as a preferred method of choice for the WSS evaluation, that WSS in the ascending aorta reaches critically high values before TAVI and is substantially reduced post-TAVI (maximum values of time-averaged WSS: 31.5 vs. 6.26 Pa for pre- vs. post-TAVI examinations, respectively). If this is true for the majority of severe AS patients referred for TAVI, the exaggerated WSS before TAVI may underline the attenuated HR hypoxic response, which is partially restored after TAVI due to the reduction of WSS in the aorta.

### 4.3. Relations Between Pre- vs. Post-TAVI Changes in Hypoxic Responses and the Changes in Hemodynamic Variables

Although the study design does not permit firm conclusions about the mechanism of the influence of TAVI on the peripheral chemoreflex or the potential role of hemodynamic factors (particularly WSS), the observed pattern of correlations partially corroborates the conceptual framework identifying reduced WSS as a key driver of peripheral chemoreceptor hypersensitivity. Specifically, a strong association between the increases in both the aortic valve area and the HR response to hypoxia (Spearman’s R = 0.8) provides substantial support for this explanation. Relieving the obstruction of the aortic inlet causes a decrease in blood flow velocity [67]. Given that blood flow velocity is proportional to WSS, a reduction in blood flow through the valve after TAVI results in decreased WSS in the aorta, and theoretically, decreased expression of the shear-stress-sensitive factor KLF2 in the aortic bodies, which is crucial for maintaining their sensitivity.

Nevertheless, the question remains as to why other hemodynamic measures did not follow the pattern revealed for the aortic valve area and HR response. A possible explanation emerges from the computational procedures. The aortic valve area is calculated using velocity–time integrals of the left ventricular outflow tract and transaortic flow [68] and therefore represents the average value for one cardiac cycle. On the contrary, the peak aortic jet velocity is derived from the single highest point of the Doppler signal envelope during systole. In light of this, the aortic valve area appears to be a better surrogate of the average blood flow velocity/WSS throughout the entire cardiac cycle than the peak aortic jet velocity. Similarly, the mean aortic valve gradient seems to be a worse surrogate of blood flow velocity/WSS than the aortic valve area due to the paradoxical low-flow, low-gradient phenotype of AS, constituting 10–25% of the severe AS population [69,70]. Nevertheless, both peak aortic jet velocity and mean aortic valve gradient, when plotted against the HR response, reveal a non-random (although statistically non-significant) pattern in our population (see supplementary Figures S1 and S2).

Furthermore, no relationship was found between changes in the LVEF and HR response. Although LVEF indeed improved after TAVI, the magnitude of the increase (~5%) might be too small to affect the chemoreceptor activity directly. Ding et al. [23,24] have demonstrated that a 35–40% reduction in CABF is required to promote peripheral chemoreceptor hyperactivity in rabbits. Nevertheless, it must be emphasized again that, according to our results, peripheral chemoreflex sensitivity in terms of HR response is suppressed in severe AS before TAVI and partially restored after TAVI, and is likely to be driven by a TAVI-dependent reduction in WSS in the aorta and not the modest improvement in LVEF.

Previous studies provided discrepant results on the effect of TAVI on LVEF. However, it seems that TAVI indeed improves LVEF, at least in patients with preoperative low ejection fractions [71,72]. About 30% of patients in the current study had a baseline LVEF < 40% (eight out of twenty-six patients). LVEF increased after TAVI in 10 patients (39%).

A lack of significant correlations between changes in HVR and changes in hemodynamic factors (all *p* > 0.12) following TAVI suggests that the surgery has a negligible effect on carotid bodies.

### 4.4. Severe AS Treated with TAVI as a Human Model of PCh Function Under Low- vs. High-Blood-Flow and/or WSS Conditions

Our study did not provide a definitive answer regarding the potential scientific utility of severe AS treated with TAVI as a human model of PCh function under conditions of altered flow/WSS. The analyses of ventilatory and cardiac components of the peripheral chemoreflex provide conflicting results in this context. Contrary to our predictions, HVR in the examined group was not augmented before TAVI and did not change following the surgery. Given that bilateral carotid body resection in HF was shown to abolish the ventilatory responsiveness to hypoxia [5,8], it may be concluded that the effect of severe AS on the carotid bodies is negligible. Traditionally, the augmented ventilatory response to PCh provocation is used to identify patients displaying PCh hypersensitivity [12,13,15,16]. The clinical or diagnostic significance of the ventilatory reflex appears to be more accentuated than the hemodynamic responses (including the HR response) despite the substantial discrepancy between the ventilatory and sympathetic components of the PCh reported recently in healthy subjects [73] and patients with hypertension and obstructive sleep apnea [74]. Nevertheless, in this context, it does not seem justified to further develop and utilize our model.

On the other hand, however, we found hypoxic HR responsiveness to be suppressed in our group before TAVI and significantly enhanced after the surgery. Although such results are in contrast to original predictions stemming from the animal studies by Ding et al. [23,24], they actually fit perfectly with the most recent, detailed studies on WSS in severe AS, which show that WSS in the ascending aorta before TAVI reaches values that are 5-fold higher than the values recorded after TAVI. A robust correlation between changes (pre- vs. post-TAVI) in the aortic valve area and HR slope provides further support for this explanation. Further studies employing WSS assessment are needed to establish the putative usefulness of severe AS treated with TAVI as a human model for studying the aortic chemoreceptor function in relation to regional WSS.

## 5. Limitations

Other differences between the animal model of chemoreceptor hypoperfusion, as described by Ding et al. [23,24], and a human pathophysiological model presented herein merit comment. First, Ding et al. investigated the development and progression of chemoreceptor hyperactivity following CABF reduction but did not test directly, in the same group of animals, whether the subsequent release of carotid artery occlusion would restore chemosensitivity. In contrast, our study is based on an assumption that chemoreceptor hypersensitivity is reversible and may improve in response to CABF increase. This assumption, however, receives considerable support from the recent study by Marcus et al. [26]. They have shown that in vivo adenoviral transfection of the shear-stress-sensitive transcription factor KLF2 to the carotid bodies in HF rabbits results in marked PCh sensitivity normalization.

Secondly, anatomical distinctions in the location and structure of the carotid/aortic bodies between animals and humans may result in different distributions of blood flow/WSS [75]. As a result, peripheral chemoreceptor function may be differentially affected by the blood flow/WSS changes in various species.

We did not measure WSS in our work. Previous studies have calculated WSS in the carotid artery based on ultrasound-derived measures and blood viscosity, as assessed in vitro with a viscometer from the venous blood sample [49]. A method utilizing cardiovascular magnetic resonance imaging and computational fluid dynamics can also be used in selected patients [66,76].

A small sample size and no control group are important limitations to our study. However, it must be considered from the perspective of this study’s aim. This is a pilot study aimed at outlining a potentially attractive field for upcoming studies—TAVI patients as a model for studying the genesis of peripheral chemoreceptor hypersensitivity. Regarding the lack of a control group, our results have referred to a number of previous studies utilizing the same method (transient hypoxia) in healthy controls aged ≥ 50 years. Some of these studies were conducted in our center, using exactly the same methodology. Given that collecting a control group of healthy subjects of an age similar to that of our AS patients (mean: 77 years) would be extremely difficult, we have decided to use data from the literature instead.

Recent studies indicate that the ventilatory response to acute hypoxia does not predict the sympathetic responsiveness of the peripheral chemoreflex [73,74]. Therefore, we cannot be certain that the sympathetic component of the peripheral chemoreflex was not altered in our study, despite the fact that the ventilatory component was relatively unchanged in most of our patients. Furthermore, the phasic sensitivity of the peripheral chemoreceptors does not necessarily resonate with their tonic activity (tonicity); therefore, testing both chemoreceptor sensitivity and tonicity is recommended [77].

## 6. Clinical Significance

Elucidating the role of altered blood flow/WSS in driving peripheral chemoreceptor function in humans carries substantial and far-reaching clinical implications in several domains, including identifying patients at risk of developing heightened chemosensitivity, as well as modifying chemosensitivity. The former area could utilize the calculation of carotid WSS or the evaluation of surrogate measures (i.e., aortic valve area or intima-media thickness). The latter area could result in developing a new, minimally invasive alternative to currently tested techniques for peripheral chemoreflex deactivation, namely surgical carotid body resection/ablation [8,15] or the pharmacological antagonism of carotid P2X3 receptors [21,78]. This could take the shape of a small-sized stent, increasing carotid body perfusion and/or WSS in that region.

## 7. Conclusions

Several novel observations have been made in the current study. Peripheral chemoreceptor function has been evaluated for the first time in the setting of severe AS. Well-preserved HVR, along with no change in HVR post-TAVI, calls into question the rationale for studying severe AS as a human model of PCh hypersensitivity.

On the other hand, the HR response to hypoxia in the study population before TAVI appeared to be reduced, and the surgery produced a significant increase in the hypoxic HR response, suggesting that severe AS treated with TAVI may be a useful model of hemodynamic factors shaping the cardiac component of the peripheral chemoreflex. A reduction in WSS in the aorta after TAVI provides a plausible explanation congruent with the current understanding of the role of WSS and the shear-stress-sensitive transcription factor KLF2 in generating PCh hypersensitivity, although neither WSS nor KLF2 have been evaluated in our study. Furthermore, the observations regarding HR response to hypoxia should be interpreted with caution given the extremely small sample size (N = 10).

## Figures and Tables

**Table 1:** Baseline demographic and clinical characteristics of the examined patients.

	All Patients (N = 26)
Male, n (%)	13 (50)
Age, years	77 ± 6
Body mass index, kg/m^2^	29.1 ± 5.1
HF, n (%)	13 (50)
Atrial fibrillation, n (%)	9 (35)
Coronary artery disease, n (%)	11 (42)
Previous myocardial infarction, n (%)	2 (8)
Hypertension, n (%)	22 (85)
Diabetes mellitus, n (%)	13 (50)
Chronic obstructive pulmonary disease, n (%)	4 (15)
Chronic kidney disease, n (%)	7 (27)
Therapies, n (%)	
Beta-blocker	24 (92)
ACE-I/ARB/ARNI	18 (69)/2 (8)/2 (8)
MRA	15 (58)
SGLT2i	4 (15)
Loop diuretics	16 (62)
Thiazides	4 (15)
CCB	10 (38)
Statin	24 (92)

Data are presented as number and percentage or mean ± SD; HF, heart failure; ACE-I, angiotensin-converting enzyme inhibitor; ARB, angiotensin receptor blocker; ARNI, angiotensin receptor/nephrilysin inhibitor; MRA, mineralocorticoid receptor antagonist; SGLT2i, sodium glucose co-transporter type 2 inhibitor; CCB, calcium channel blocker.

**Table 2:** Effect of TAVI on hemodynamic, respiratory, and blood parameters for the examined patients.

	N	Before TAVI	After TAVI	*p*-Value
*Ultrasound measurements*				
Aortic valve area, cm^2^	26	0.7 [0.5–0.9]	1.9 [1.7–2.0]	<0.001
Mean aortic valve gradient, mmHg	26	50 ± 15	11 ± 4	<0.001
Peak aortic jet velocity, m/s	26	4.4 ± 0.7	2.2 ± 0.4	<0.001
LVEF, %	26	50 ± 15	55 ± 11	0.020
CABF ^†^, mL/min	18	576 ± 166	578 ± 158	0.933
*Respiratory and hemodynamic parameters at rest*				
$\dot{V}$, L/min	26	11 ± 3	11 ± 2	0.388
HR, bpm	10	62 ± 12	63 ± 11	0.825
SBP, mmHg	10	113 ± 23	128 ± 29	0.210
MAP, mmHg	10	74 ± 12	83 ± 17	0.112
SVR, dyn × s/cm^5^	10	1411 ± 251	1596 ± 341	0.217
Cardiac output, L/min	10	4.4 ± 0.7	4.4 ± 0.9	0.907
*Blood parameters*				
Hemoglobin, g/dL	26	12.9 ± 1.2	13.3 ± 1.2	0.047
Hematocrit, %	26	38.4 ± 3.9	40.1 ± 3.6	0.017
Creatinine, mg/dL	26	1.00 [0.89–1.25]	0.97 [0.83–1.19]	0.043
Sodium, mmol/L	26	140.4 ± 2.5	140.7 ± 2.4	0.571
Potassium, mmol/L	26	4.36 ± 0.49	4.53 ± 0.62	0.079
NT-proBNP, pg/mL	26	1925 [1278–3575]	698 [422–1005]	0.016

Data are presented as mean ± SD or median with IQR where appropriate; *p*-value for before-TAVI vs. after-TAVI comparisons with the Student’s paired *t*-test or Wilcoxon test; ^†^ data from 17 patients; LVEF, left ventricular ejection fraction; CABF, carotid artery blood flow; $\dot{V}$, minute ventilation, HR, heart rate; SBP, systolic blood pressure; MAP, mean arterial pressure; SVR, systemic vascular resistance; NT-proBNP, N-terminal pro-B-type natriuretic peptide.

**Table 3:** Correlations between the pre-post TAVI change (Δ) in peripheral chemoreceptor function and the pre- and post-TAVI changes in hemodynamic parameters.

	Δ HVR, L/min/SpO_2_%	Δ HR Slope, bpm/SpO_2_%
Δ Aortic valve area, cm^2^	−0.14 (*p* = 0.499), N = 26	−0.80 (*p* = 0.006), N = 10
Δ Mean aortic valve gradient, mmHg	−0.15 (*p* = 0.477), N = 26	−0.43 (*p* = 0.214), N = 10
Δ Peak aortic jet velocity, m/s	−0.30 (*p* = 0.137), N = 26	−0.26 (*p* = 0.467), N = 10
Δ LVEF, %	−0.09 (*p* = 0.667), N = 26	−0.03 (*p* = 0.933), N = 10
Δ CABF, mL/min	−0.01 (*p* = 0.958), N = 18	−0.14 (*p* = 0.736), N = 10

R Spearman’s rank correlation coefficient values are presented with *p*-values; LVEF, left ventricular ejection fraction; CABF, carotid artery blood flow; HVR, hypoxic ventilatory response (as assessed with the transient hypoxia test), HR slope, heart rate response (as assessed with the transient hypoxia test).

## Data Availability

The original contributions presented in the study are included in the article, further inquiries can be directed to the corresponding author.

## Supplementary Materials

The following supporting information can be downloaded at: https://www.mdpi.com/article/10.3390/biomedicines13030611/s1, Figure S1. Scatter plot illustrating the relation between pre- vs. post-surgery change in HR slope and the change in peak aortic jet velocity.; Figure S2. Scatter plot illustrating the relation between pre- vs. post-surgery change in HR slope and the change in mean aortic valve gradient.
