# Supplementary material for: Hemodynamic Factors Driving Peripheral Chemoreceptor Hypersensitivity: Is Severe Aortic Stenosis Treated with Transcatheter Aortic Valve Implantation a Valuable Human Model?
Source: Biomedicines. 2025 Mar 3;13(3):611. doi: 10.3390/biomedicines13030611 (PMC11940327; doi:10.3390/biomedicines13030611)

Figure S1. Scatter plot illustrating the relation between pre- vs. post-surgery change in HR slope and the change in peak aortic jet velocity.

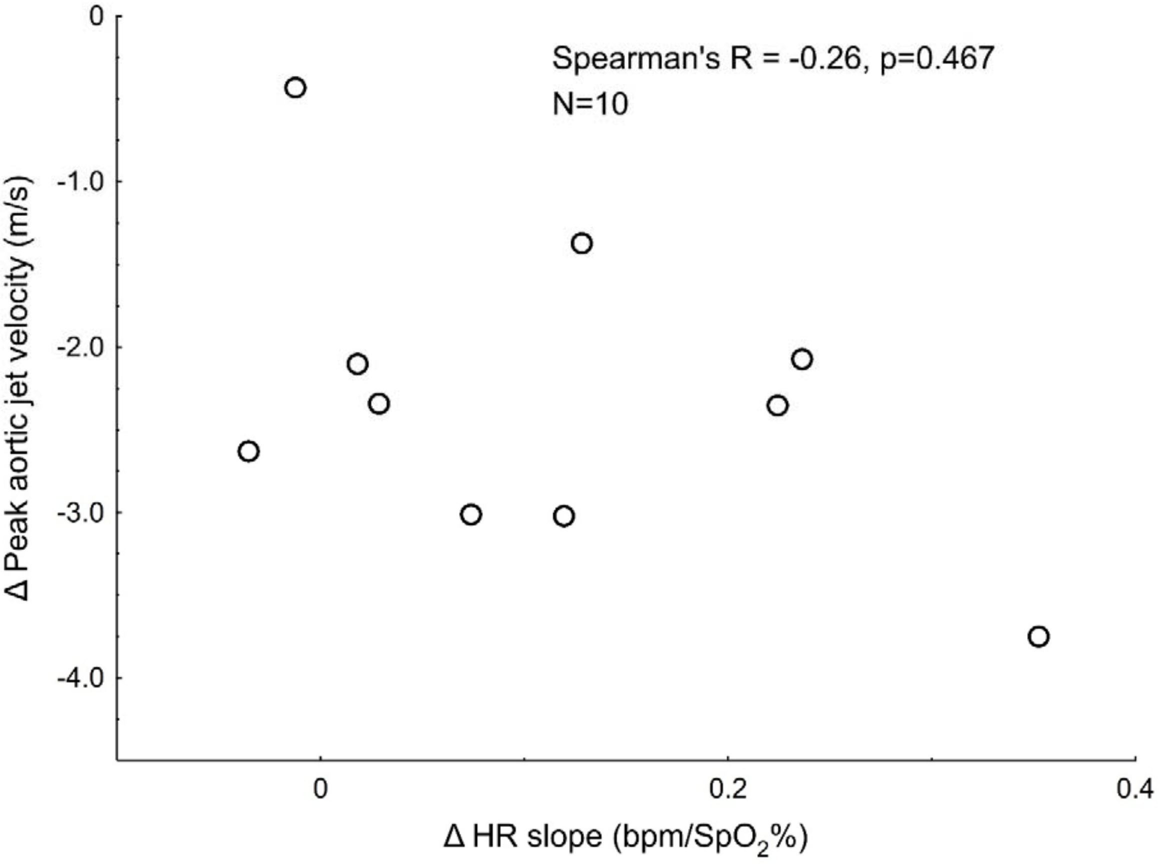

Figure S2. Scatter plot illustrating the relation between pre- vs. post-surgery change in HR slope and the change in mean aortic valve gradient.

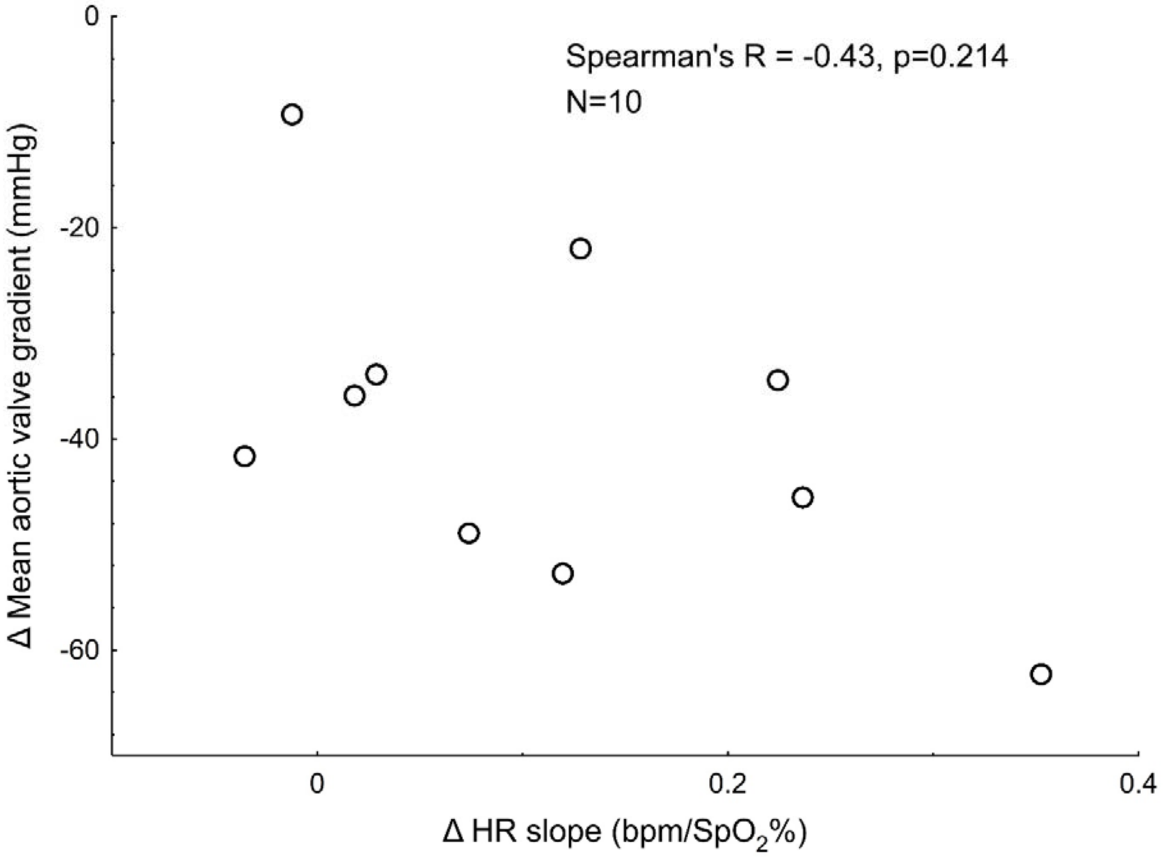

Supplement: Supplementary file 1 [file biomedicines-13-00611-s001.zip › biomedicines-3433842-supplementary.pdf]
